# Refeeding Syndrome With Hyperemesis Gravidarum: A Case Report

**DOI:** 10.7759/cureus.27178

**Published:** 2022-07-23

**Authors:** Amit Ramrattan, Saeeda Mohammed, Muhammad Rahman

**Affiliations:** 1 Internal Medicine, Port-of-Spain General Hospital, Port of Spain, TTO; 2 Gastroenterology, Port-of-Spain General Hospital, Port of Spain, TTO

**Keywords:** obstetrics, rhabdomyolysis, hypophosphatemia, hyperemesis gravidarum, refeeding syndrome

## Abstract

Refeeding syndrome refers to a clinical condition whereby biochemical abnormalities such as hypokalemia, hypomagnesemia, and mainly hypophosphatemia occur upon recommencement of feeding, typically in malnourished catabolic patients. A 36-year-old female presented at 30 weeks gestation with hyperemesis gravidarum. During this admission, the patient developed transaminitis with low platelets prompting suspicion of hemolysis, elevated liver enzymes low platelets (HELLP) syndrome, but the concurrent findings of severe hypophosphatemia and rhabdomyolysis were not in favor of HELLP syndrome but that of refeeding syndrome. These clinical entities proved to be a diagnostic dilemma but, ultimately, the patient was managed under refeeding syndrome. The multidisciplinary approach amongst gastroenterology, internal medicine, obstetrics, hematology, and dietetics departments led to the slowing up of the titration of caloric feeds, which ultimately led to full recovery.

## Introduction

Refeeding syndrome refers to a clinical condition whereby biochemical abnormalities such as hypokalemia, hypomagnesemia, and mainly hypophosphatemia occur upon recommencement of feeding, typically in malnourished catabolic patients. Hypophosphatemia being the hallmark of the condition, it can have a barrage of clinical manifestations that can be life-threatening if not sought for or managed adequately, highlighting the need for increased awareness and a multidisciplinary approach to this condition. Hyperemesis gravidarum (HG) is the persistence of nausea and vomiting in pregnancy, and apart from being a debilitating obstetric condition, it makes a patient susceptible to refeeding syndrome if severe and prolonged.

We present a case of a 36-year-old patient Gravida 4 Para 3+0 at 30+1/40 weeks of gestation who developed refeeding syndrome from severe HG, which proved to be a diagnostic dilemma after the patient developed unforeseen complications. The patient presented for HG. During this admission, she developed transaminitis with low platelets prompting suspicion of hemolysis, elevated liver enzymes low platelets (HELLP) syndrome, but the concurrent findings of severe hypophosphatemia and rhabdomyolysis pointed towards refeeding syndrome. These clinical entities proved to be a diagnostic dilemma; however, ultimately, the patient was managed under refeeding syndrome, which ultimately led to full recovery.

## Case presentation

A 36-year-old Gravida 4 Para 3+0, with three previous pregnancies being normal spontaneous vaginal deliveries, presented at 30+1/40 weeks of gestation with vomiting since the first trimester and significant weight loss of more than 40 lbs compared to her pre-pregnancy weight. She had multiple admissions to other primary and tertiary care institutions with these symptoms, which were treated with intravenous fluids and anti-emetics prior to discharge. On presentation to our institution, she was admitted to the Obstetrics unit and referrals were made to the Gastroenterology and Medicine units due to persistent, severe symptoms. 

The patient had a suboptimal blood pressure of 106/68 mmHg, heart rate of 92 beats per minute, respiratory rate of 18 breaths per minute, and a temperature of 36˚C. On examination, the patient was noted to have oral candidiasis, but the rest of her examination was unremarkable apart from a gravid uterus with fundus at the level of the umbilicus in keeping with the gestational age.

On admission, the investigations were as follows; renal function test showed sodium 138 mmol/L, potassium 1.8 mmol/L, blood urea nitrogen 6mg/dl, creatinine 0.56mg/dl, phosphate 2.25mg/dl, calcium 8.7mg/dl. Complete blood count showed a white cell count of 6.6 x109/L, hemoglobin 11.8g/L, mean corpuscular volume 88.6fL, and platelet 119 x109/L. Liver enzymes were mildly elevated with aspartate aminotransferase (AST) 53U/L and albumin low at 2.9g/dL. Thyroid stimulating hormone was 0.59 uIU/ml which was normal and HIV Rapid test, Hepatitis B, Hepatitis C, and venereal disease research laboratory (VDRL) tests were negative. Urinalysis on admission showed 3+ ketones with trace blood and protein. Abdominal ultrasound imaging showed normal appearances of the liver, gallbladder, common bile duct, kidneys, ureters, and bladder. Pelvic ultrasound done during her presentation as well as on discharge showed normal fetal growth parameters in addition to a normal resistive index of the umbilical artery.

Upon admission, the patient was commenced on intravenous fluids comprising dextrose 5% and normal saline with potassium chloride supplementation and vitamin B-complex. This patient underwent an upper endoscopy, which was normal and a nasojejunal tube was placed during the procedure to facilitate enteral feeding. Due to the patient’s severe hypokalemia of 1.8mmol/L, she was transferred to the High Dependency Unit for cardiac monitoring. Her nasojejunal feeds, after consultation with the dietitian, were commenced at half her required daily intake starting at 430 kcal at the time to mitigate the risk of developing refeeding syndrome. On day four of admission, the patient’s phosphate was noted to have precipitously dropped to as low as 0.4 mg/dl after which she experienced muscle cramps and weakness of her thighs along with hyperreflexia of her upper limbs. At this time, her urinalysis showed 3+ blood, AST had risen to 2557 U/L (reference range 5-40 U/L) and LDH 1717 U/L (reference range 135-225 U/L). Platelet count was 96 x109/L and an International Normalized Ratio was 1.4. A blood smear revealed some schistocytes, but serial blood films failed to reproduce these findings. Mid-stream urine testing showed 10-12 red blood cells/high power field. Neither urinary myoglobin nor serum creatine kinase was not performed due to non-availability. Out of institution creatine kinase done after two days was 2079 U/L (reference range 29-168 U/L) and by this time her AST had already dropped to 400 U/L and LDH 351 U/L. An autoimmune screen done at the time of this transaminitis showed normal C3 and C4 levels with a negative Rheumatoid Factor. antinuclear antibody (ANA) testing was unavailable. A trend in the biochemistry results can be seen in Table 1.

**Table 1:** Electrolytes and liver enzymes trend

	Day 1	Day 2	Day 4	Day 5	Day 6	Day 8	Day 10	Day 14	Reference Range
Potassium	1.8	2.2	2.8	2.8	2.8	3.1	3.4	3.5	3.5-5.1 mmol/L
Phosphorus	2.25	1.09	0.4	0.45	0.58	1.25	1.53	3.11	2.7-4.5 mg/dl
Magnesium	1.87	1.85	1.71	1.65	1.54	2.07	2.05	1.71	1.7-2.55 mg/dl
Creatinine	0.53	0.56	0.52	0.39	0.4	0.41	0.43	0.3	0.5-0.9 mg/dl
Aspartate transaminase	53	59	82	2879	2553	400	112	39	5-38 U/L
Lactate dehydrogenase	267	280		1671	1092	351		303	135-214 U/L
Direct bilirubin	0.7	0.5	0.4	1.9	2.3	1.6	0.8	0.6	0-0.4 mg/dl
Indirect bilirubin						0.2	0.2	0.1	0.1-1 mg/dl
International normalized ratio	1.2	1.94		1.86		1.25			
Hemoglobin	11.8	10.4	10.3	10.1	9.3	9.0	9.1	8.4	11-14 g/L
Platelets	119	119	96	138	148	145	167	140	150-400 10x9/L

With the evolution of severe hypophosphatemia, intravenous dextrose was discontinued, and her calories were further decreased to a quarter of her requirements by weight. There was, however, no intravenous phosphate replacement therapy available on the island. A trial of sodium phosphate enema failed as the patient could not tolerate it. She then had to be commenced on oral phosphate supplementation given via her nasojejunal tube. During this time, there was a multidisciplinary meeting with Obstetricians, Medicine, Gastroenterology, and Neonatology. The patient was commenced on dexamethasone to promote fetal lung maturity in the event of an emergency delivery as hemolysis, elevated liver enzymes low platelets (HELLP) syndrome was a differential diagnosis.

Catering to the caloric requirements of both mother and fetus without causing further decrements of the phosphate levels proved challenging but after a multidisciplinary effort, slow up-titration of the calories was done successfully. Phosphate levels were closely followed, and progressive resolution allowed slow up-titration of the caloric intake. After three weeks of hospitalization with nasojejunal feeds and oral phosphate supplementation, the patient’s electrolytes, liver enzymes, creatine kinase, platelets, proximal myopathy, and hyperreflexia, everything normalized. The nasojejunal tube was removed and oral feeds were commenced and tolerated before she was discharged from the hospital.

## Discussion

Refeeding syndrome is a rare complication of HG with only few case reports in publication. Shifts in electrolyte and fluid balance in malnourished patients upon recommencement of feeding, both enterally and parenterally [1] causes hypokalemia, hypomagnesemia, and thiamine deficiency, in addition to hypophosphatemia, which is the hallmark of this condition and is associated with morbidity and mortality [2]. Severe HG, requiring hospitalization, in itself, only occurs in 0.3-2% of pregnancies [3]. A general definition for refeeding syndrome is not available; however, it is usually associated with the development of severe electrolyte abnormalities in the first days after commencement of feeding, which may be enteral or parenteral. The metabolic disturbances can lead to potentially fatal outcomes if not identified early.

Normal physiology is altered during periods of prolonged fast/starvation. In a response to low levels of glucose, insulin levels fall and glucagon levels rise. There is a shift to gluconeogenesis. Eventually, in a prolonged fasting state, fats and proteins become the primary source of energy. There is a loss of electrolytes, especially phosphorus, potassium, and magnesium [4]. There may also be depletion of micronutrients and vitamins as well, particularly thiamine.

During refeeding, as glucose is reintroduced, fat metabolism slows down and glucose metabolism resumes. The resulting hyperglycemia causes spikes in insulin, which activates the Na+ - K+ ATPase transporters present on cells leading to shift of potassium and water into cells. Sudden shifts of potassium and phosphate from the in extracellular to intracellular space causes a precipitous fall in serum levels. In this case, the serum phosphorus, potassium, and magnesium were already low prior to feeding. The introduction of parenteral feeding combined with the use of dextrose intravenous fluid likely precipitated the refeeding syndrome.

Rhabdomyolysis, as a result of severe hypophosphatemia (serum phosphate <1.0 mg/dL), was anticipated. The early identification of rhabdomyolysis was important in treatment modification (increased intravenous fluids and phosphate correction). This may have prevented complications of rhabdomyolysis such as acute kidney injury. Rhabdomyolysis in the presence of hypophosphatemia has been established in a 1978 study done on dogs fed a low phosphate and low calorie diet [5]. There are also several human case reports and studies, particularly in patients with alcoholism, showing similar findings [6,7]. 

The patient showed evidence of hemolysis with schistocytes on blood film, rising lactate dehydrogenase (LDH), and mild hyperbilirubinemia. Hemolysis associated with hypophosphatemia is rare and has been reported in the literature, especially when there is a sudden decline in phosphate levels, like that seen in refeeding syndrome. Phosphorus is needed to manufacture erythrocyte ATP. Low levels of red cell ATP is associated with loss of red cell deformability which causes loss of the normal biconcave shape. This leads to the formation of rigid spherocytes or echinocytes, which are prone to intravascular hemolysis [8,9]. Hemolysis has been found to be reversible with correction of the serum phosphate levels. 

Given the evidence of hemolysis, elevation in liver enzymes and low platelet count, there was a consideration that the patient may have developed HELLP syndrome [10]. However, the repeat blood film showed no evidence of hemolysis and the findings were better attributed to refeeding syndrome. The patient did not have hypertension or proteinuria that may accompany HELLP syndrome. Antepartum steroid prophylaxis for fetal lung maturity was given after a decision by the multidisciplinary team in case of preterm delivery. As the patient was only at 30 weeks of gestation, this conservative approach was undertaken with close monitoring of the fetus and mother in the high dependency unit. There was eventual resolution of electrolyte abnormalities and transaminitis as well as thrombocytopenia. The patient was able to have a healthy baby via normal, spontaneous vaginal delivery at term. 

Hypophosphatemia can cause other hematological abnormalities such as thrombocytopenia (rarely) and platelet dysfunction [11]. This patient developed thrombocytopenia, which resolved as the phosphate levels improved. Electrolyte and fluid abnormalities should be corrected, and the patient should have continuous cardiac monitoring to observe for any potential cardiac arrhythmias [12,13]. Cardiac arrhythmias are the most common cause of death in these patients.

Due to a lack of institutional policies in the management of refeeding syndrome, a multidisciplinary approach is important in positive outcomes for patients [14]. Patients at risk should have serial electrolyte measurements done prior to and upon initiation of feeding. Vitamin supplementation, particularly thiamine, should be started first to prevent Wernicke’s encephalopathy. Caloric intake should not exceed 50% of the daily requirement and should be increased slowly

Implementation of a simple, validated malnutrition screening tool should be done for all cases of HG to identify persons at risk and therefore avoid the significant morbidity or mortality associated with refeeding syndrome. There is need for increased physician awareness of this condition [15]. 

## Conclusions

This case highlights a classic case of refeeding syndrome with legion complications from severe hypophosphatemia including rhabdomyolysis, thrombocytopenia, and proximal myopathy. One should always consider a malnourished patient to be at risk for developing a refeeding syndrome and take measures to avoid its occurrence. A multidisciplinary approach is essential in managing patients at risk for developing a refeeding syndrome and all parties involved must communicate well and implement a strategic plan in managing these patients. HG should also be considered a risk factor for the development of refeeding syndrome as shown in this case.

## References

[1] Boland K, Solanki D, O’Hanlon C (2013). IrSPEN Guideline Document No. 1: Prevention and Treatment of Refeeding Syndrome in the Acute Care Setting. https://www.irspen.ie/wp-content/uploads/2014/10/IrSPEN_Guideline_Document_No1.pdf.

[2] Marinella MA (2003). The refeeding syndrome and hypophosphatemia. Nutr Rev.

[3] Goodwin TM (2008). Hyperemesis gravidarum. Obstet Gynecol Clin North Am.

[4] McCray S, Parrish CR (2016). Refeeding the malnourished patient: lessons learned. Pract Gastro.

[5] Knochel JP, Barcenas C, Cotton JR, Fuller TJ, Haller R, Carter NW (1978). Hypophosphatemia and rhabdomyolysis. J Clin Invest.

[6] Singhal PC, Kumar A, Desroches L, Gibbons N, Mattana J (1992). Prevalence and predictors of rhabdomyolysis in patients with hypophosphatemia. Am J Med.

[7] Amanzadeh J, Reilly RF Jr (2006). Hypophosphatemia: an evidence-based approach to its clinical consequences and management. Nat Clin Pract Nephrol.

[8] Jacob HS, Amsden T (1971). Acute hemolytic anemia with rigid red cells in hypophosphatemia. N Engl J Med.

[9] Melvin JD, Watts RG (2002). Severe hypophosphatemia: a rare cause of intravascular hemolysis. Am J Hematol.

[10] Barton JR, Sibai BM (2004). Diagnosis and management of hemolysis, elevated liver enzymes, and low platelets syndrome. Clin Perinatol.

[11] Khan LU, Ahmed J, Khan S, Macfie J (2011). Refeeding syndrome: a literature review. Gastroenterol Res Pract.

[12] Mehanna HM, Moledina J, Travis J (2008). Refeeding syndrome: what it is, and how to prevent and treat it. BMJ.

[13] McKnight CL, Newberry C, Sarav M, Martindale R, Hurt R, Daley B (2019). Refeeding syndrome in the critically ill: a literature review and clinician's guide. Curr Gastroenterol Rep.

[14] Jo HJ, Shin DB, Koo BK, Ko ES, Yeo HJ, Cho WH (2017). The impact of multidisciplinary nutritional team involvement on nutritional care and outcomes in a medical intensive care unit. Eur J Clin Nutr.

[15] Janssen G, Pourhassan M, Lenzen-Großimlinghaus R (2019). The refeeding syndrome revisited: you can only diagnose what you know. Eur J Clin Nutr.

